# Childhood Mortality Due to Drowning in Rural Matlab of Bangladesh: Magnitude of the Problem and Proposed Solutions

**Published:** 2007-09

**Authors:** Anwarul Iqbal, Tahmina Shirin, Tahmeed Ahmed, Sirajuddin Ahmed, Noor Islam, Arif Sobhan, A.K. Siddique

**Affiliations:** 1 ICDDR, B, GPO Box 128, Dhaka 1000, Bangladesh; 2 National Institute of Kidney Diseases and Urology, Sher-e-Bangla Nagar, Dhaka 1207, Bangladesh

**Keywords:** Awareness, Causes of death, Child mortality, Drowning, Infant mortality, Interventions, Observational studies, Bangladesh

## Abstract

Drowning is an important cause of mortality among children in rural Bangladesh. Children aged 1–4 year(s) are at a high risk of death from drowning. Although deaths of children due to drowning in Bangladesh are acknowledged as an important cause of death, little effort has been made to address the issue of preventing deaths from this cause. This study has attempted to describe the problem and suggests possible prevention strategies, which may contribute to reducing childhood mortality from drowning. Data presented in this study were collected from Matlab where ICDDR, B has been maintaining a demographic surveillance since 1966. During the study period from 1985 to 2000, 989 deaths from drowning were reported, of which 796 (80.5%) were children in the age-group of 1–4 year(s), 48 (4.8%) were in the age-group of less than one year, and 145 (14.7%) in the age-group of 5–19 years. During 1985–2000, death rate per 1,000 children due to all causes among children of 1–4-year age-group decreased appreciably from 20.7% to 5.2%, while drowning-related deaths did not. Forty-five percent (n=359) of drowning-related deaths occurred in ponds, 16.8% (n=134) in ditches, 8.1% (n=64) in canals, and 4.4% (n=35) in rivers. The sites of more than 25% of drowning-associated deaths were not recorded. Analysis of seasonal variation revealed that most deaths due to drowning occurred during April-October, i.e. mostly during the monsoon months. It was also observed that the majority (67%) of mothers of victims had no formal education. Deaths due to drowning were mostly associated with children aged 1–4 year(s) and were 20% more common among boys than among girls (odds ratio=1.2, 95% confidence interval 1.04–1.38, p<0.012). The paper recommends some interventions to reduce the number of deaths due to drowning in rural Bangladesh, which include: (a) increasing awareness among mothers and close family members about the risk of drowning, (b) door-fencing, and (c) filling of unused ditches and water holes around households.

## INTRODUCTION

Effective therapeutic and preventive measures against different infectious diseases have remarkably reduced child mortality. Unintentional injuries, including drowning, continue to be one of the leading causes of childhood mortality among children. It was estimated that nearly half a million deaths were due to drowning in 1998 globally, 57% of which occurred among children (1,2). Results of studies in different countries have shown that drowning is one of the leading causes of mortality among children (3-6). Data from different countries indicate that children aged 0–4 year(s) are at an increased risk of drowning (4,7–14). Although in developed countries, some data regarding drowning-related deaths do exist, they are under-reported (15), and data on drowning-related deaths are scarce in many less-developed and poorer countries.

The rate of child mortality in Bangladesh is still high compared to other countries in Asia (16). In Bangladesh, it has declined from 20 per 1,000 persons in 1983 to nearly 8 per 1,000 persons in 1995. This has been attributed to various intervention programmes and other preventive measures undertaken at the national level against infectious diseases and malnutrition. However, deaths from environmental hazards, including drowning, have not been adequately studied and addressed in Bangladesh (17-20). In developed countries, children of 1 to 4-year age-group are most likely to drown in swimming pools, particularly in residential areas (21-25).

Different studies in the USA proposed several preventive strategies, such as pool-fencing, installation of pool-alarm and telephone, and cardiopulmonary resuscitation in the case of near-drowning, along with parent's awareness (21,26–30). Although there are a few articles on drowning-related deaths in the middle- and low-income countries, results of these studies showed that many cases of drowning took place in buckets (15,30), tubs, ponds, and unprotected wells (5).

A study in Bangladesh explored the epidemiological profile of deaths of children due to drowning, community experience about near-drowning, and community perceptions regarding potential interventions against drowning. The study included the findings of two empirical datasets: the source of one dataset was the nationally-representative Bangladesh Demographic and Health Survey (BDHS) 1996–1997, and the other one was the Health and Demographic Surveillance System (HDSS, Matlab) of ICDDR, B from 1982 to 1995 (31). In this study, it was observed that application of appropriate cardiopulmonary resuscitation techniques in the cases of near-drowning was not adequate. However, the community perception about the potential intervention against drowning of children revealed a common response in both national and the Matlab surveys, which was to increase maternal vigilance. Some respondents in the BDHS suggested fencing of ponds, while the common response in Matlab was to attach a bell to the child's body so that the mother could hear the movement of the child (31).

In this study, we have attempted to describe some aspects of epidemiology of drowning in rural Bangladesh and possible interventional measures.

**Figure:**
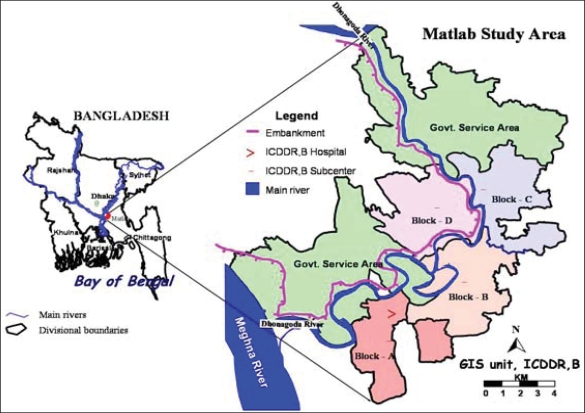


## MATERIALS AND METHODS

### Data collection

This observational study was conducted using an organized dataset collected by the HDSS from 1985 to 2000 in Matlab, a field station of ICDDR, B, located 45 km southeast of capital city Dhaka (Map).

The study included children, aged 0–19 year(s), who died from unintentional or accidental drowning. A verbal autopsy questionnaire was used for each case for finding out the cause of death. Field Research Assistants (FRAs) collected information which was verified by Medical Assistants in Matlab. Verbal autopsy, an epidemiological tool, is widely used for ascribing causes of death by interviewing bereaved relatives of the deceased who were not under medical supervision at the time of death (32). All deaths of children due to drowning were subdivided into three groups: less than one year, 1–4 year(s), and 5–19 years. Children aged less than one year were excluded because they are not at a high risk as they are usually not capable of moving by themselves, and similarly children aged more than four years were excluded as they are capable of protecting themselves from different surface waters. Detailed analyses were done only for children of 1–4 year(s) age-group. For analysis, information on age, sex, sites, and the month of drowning was considered, along with the number of siblings and education of mother of the victim. ICDDR, B has been operating the HDSS in Matlab since 1966, which maintains the vital events, such as birth, death, marriage, migration, socioeconomic condition, and also periodic census (33).

### Data analysis

The SPSS software (version 7.5) and the Epi Info 2000 software were used for analyzing data. The chi-square test was used for determining the association between the dependent and the independent variables. ANOVA was used for observing the variation of means among different age-groups. Microsoft Excel 2000 was used for creating graphs.

## RESULTS

Of 989 drowning-associated deaths in Matlab upazila during 1985–2000, around 80% occurred among children of 1–4 year(s) age-group compared to 4.8% among infants and 14.7% in more than four years age-group (p<0.0001) (Table).

**Table:** Age distribution of children and adolescents who died due to drowning in Matlab, Bangladesh

	<1 year		1–4 year(s)		5–19 years		Total
Year	No.	%	No.	%	No.	%	
1985	01	1.1	71	78.9	18	20.0	90
1986	04	6.3	48	76.2	11	17.5	63
1987	03	4.3	55	78.6	12	17.1	70
1988	06	6.9	64	73.6	17	19.5	87
1989	07	10.8	49	75.4	09	13.8	65
1990	04	6.8	50	84.7	05	8.5	59
1991	04	7.0	47	82.5	06	10.5	57
1992	03	7.9	31	81.6	04	10.5	38
1993	06	10.5	43	75.4	08	14.0	57
1994	00		30	68.2	14	31.8	44
1995	03	4.9	51	83.6	07	11.5	61
1996	00		55	85.9	09	14.1	64
1997	00		39	81.2	09	18.8	48
1998	01	1.8	52	91.2	04	7.0	57
1999	00		55	98.2	01	1.8	56
2000	06	8.2	56	76.7	11	15.1	73
Total	48	4.8	796	80.5	145	14.7	989

Analysis of variance, p>0.0001

**Fig. 1:**
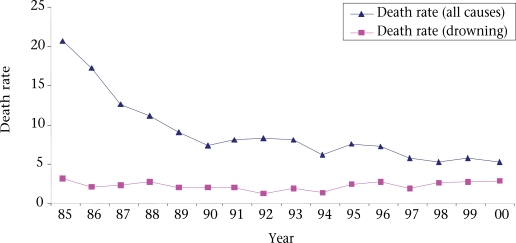
Comparison between drowning-associated death rates and rates of death due to all causes among children of 1–4 year(s) age-group in Matlab, Bangladesh

Death rate per 1,000 children due to all causes among children of 1–4 year(s) age-group decreased from 20.7% in 1985 to 5.2% in 2000. While drowning-related death rate remained almost unchanged (Fig. 1).

**Fig. 2:**
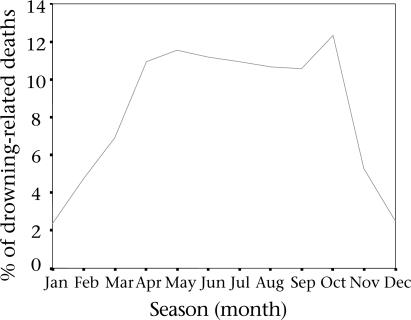
Seasonal distribution of drowning- associated deaths among children of 1–4 year(s) age-group in Matlab, Bangladesh

Regarding seasonal variation of drowning-associated deaths among children aged 1–4 year(s) in Matlab during 1985–2000, the mean death rates were 11.3%, 3.75%, and 9.8% in the monsoon (June to October), in the winter (November to February), and in the hot season (March to May) respectively. Figure 1 shows a sharp rise in drowning-associated deaths at the time of the first rainfall which continued for the whole monsoon season with two peaks in May and October, followed by a sharp decline with reduction in surface water (Fig. 2). Data relating to the sites of drowning of 1 to 4-year old children were collected from 1991 to 2000. Ponds were identified as the most common site (45.1%) for drowning, followed by ditches (16.8%), canals (8.1%), and rivers (4.4%). In the remaining 25.7% of the cases, sites of drowning could not be ascertained as these sites were not mentioned in the death records.

The total number of drowning-associated deaths was 796 among children of 1–4 year(s) age-group; of them, 440/181,822 were males and 356/176,042 were females. The death rate per 1,000 children among boys and girls was 2.4% and 2.0% respectively, the risk of drowning being 20% higher among boys (odds ratio=1.2, 95% confidence interval 1.04–1.38, p<0.012). Around 50% of 796 mothers of victims had more than three siblings, and 67% of 761 mothers of victims had no formal education.

## DISCUSSION

In this study, an attempt was made to explore the incidence and some potential risk factors of drowning-associated deaths in Matlab during 1985–2000. Age distribution of 989 dead children showed that drowning-associated deaths were significantly higher among children of 1–4 year(s) age-group compared to other age-groups. This finding is very close to observations in other countries (3,4,7–14). Accidental drowning-associated deaths occurred more commonly in children, and this might be due to behavioural patterns of children of this age-group. The overall death rate per 1,000 children deceased from 20.7% in 1985 to 5.2% in 2000. A similar trend was reported from a country-wide survey in Bangladesh (34). This downward trend in mortality among children may be due to a reduction in the occurrence of infectious diseases, which is attributed to different intervention programmes, such as immunization, management of diarrhoeal diseases, and malnutrition (35). With the gradual decline in the total death rate among 1–4 year(s) age-group from 1985 to 2000, rates of drowning-associated deaths of the same age-group remained static and, ultimately, may occupy a significant proportion of total mortality among children.

It was observed that most deaths among the target age-group occurred in ponds, followed by ditches, canals, and rivers. These findings are consistent with observations in the UK and USA (36,37). In rural Bangladesh, ponds, ditches, and, sometimes, rivers are located very close to houses, which are routinely used for household work throughout the year. As a result, children can easily roam around these sites whenever they get an opportunity, and accidents occur more in this age-group, as they are more curious, can move independently, and usually have a natural affinity for water. Living in close proximity to a water body may be considered a risk factor of drowning. Results of most studies in developed countries showed that drowning-associated deaths among children aged 1–4-year (s) occurred in swimming pools (21-25), which is not comparable with the scenario in rural Bangladesh. April to October are high-risk months for drowning, and these months cover the first rainfall and the entire monsoon season of Bangladesh. Sometimes, rainwater is enough for drowning in the case of young children.

In Matlab, around 47.4% of women have no formal education (38). In the present study, 67% of mothers of victims were illiterate, which is significantly higher. More than 50% of mothers of victims had three or more children at the time of their child's death. Lack of awareness and number of children in a family may influence unintentional drowning-associated deaths among this high-risk age-group.

In developed countries, different preventive strategies have been proposed (21,26–30). Moreover, experience shows that intervention programmes addressing injury prevention can be effective in those countries. However, in the case of developing countries, policy-makers, including health professionals, have been slow in recognizing injuries, particularly drowning-associated deaths, as a public-health problem (31). Baseline observational studies were carried out in Bangladesh regarding community perceptions about near-drowning and potential intervention against it. These studies, based on a small sample size, recommended the attachment of bell to the child's waist and the fencing of ponds or other open water bodies (31). We, however, emphasize the increasing awareness among parents and other options, including putting up temporary door-fences to keep the child inside, along with filling of unused ditches and water holes around households. Door-fencing is less costly than other alternatives, such as pond-fencing, because there are thousands of ponds and ditches in Matlab and all over the country.

As ICDDR, B has the HDSS in Matlab, which maintains all vital statistics of the assigned areas by the Community Health Research Workers (CHRWs), Health Assistants (HAs), and FRAs, an intervention programme should be carried out in Matlab. Moreover, there are two areas in Matlab: one is the intervention area which is also known as MCH-FP area, and the other one as the comparison area. The proposed intervention programme against drowning could be implemented in the MCH-FP area. During the first phase of the intervention programme, the field workers of ICDDR, B could be informed about the impact and importance of accidental drowning, then they could also be provided with the knowledge of how to prevent accidental drowning. These preventive measures may include development of awareness among the target groups, such as mothers, grandmothers, elder sisters, and heads of households, regarding deaths due to drowning. For this purpose, children should always be closely watched mainly by mothers or by any responsible person while mothers are busy with other household work. Children should never be left alone in any condition. Temporary door-fencing could be considered an alternate option to keep the child inside the room for the time being when mothers are busy with household work and other responsible persons are not available to look after the child. Unused ditches and holes should be filled up as these are the sites of a significant number of accidental drowning-associated deaths. Outcome of the intervention programme could be evaluated by comparing the incidence rate of drowning-associated deaths among children of 1–4 year(s) age-group between the MCH-FP (intervention) area and the comparison area. The effects of the intervention could also be evaluated by comparing the drowning-associated death rates in the MCH-FP area before and after implementation of the intervention.

As this study included data only on the cases of drowning of children in Matlab, there were no data on survivors matched by age, sex, socioeconomic status, and education of mothers. Therefore, no comparison could be done; as a result, a descriptive observational study was carried out. In this study, all the drowning cases were included, except those who were drowned by boat capsize. Here, the dataset also does not include time and distance of the drowning sites (ponds, rivers, ditches, and open wells) from the victim's house, and there is no separate dataset of drowning during floods.

The incidence of deaths due to drowning was significantly higher among children in the 1–4 year(s) age-group. Deaths due to other causes showed a downward trend while drowning-associated deaths remained static. Around 60% of deaths due to drowning occurred in ponds and ditches, which are situated around the victims’ households. As expected, the incidence of deaths due to drowning was higher during the monsoon. Drowning remains an important and preventable cause of childhood mortality, which requires adequate preventive strategies. An intervention programme for preventing drowning-associated deaths is proposed. This intervention is based on building awareness about the risk of drowning, along with some simple aids, such as door-fencing and filling-up of unused natural or man-made ditches and holes.

## References

[1] Crawley T (1996). Childhood injuries: Significance and prevention strategies. J Pediatr Nurs.

[2] Krug E (2002). Injury: A leading cause of the global burden of disease.

[3] Silva DT, Ruben AR, Wronski I, Stronach P, Woods M (1998). Excessive rates of childhood mortality in the northern territory. 1985–94. J Paediatr Child Health.

[4] Calder RA, Clay CY (1990). Drowning in florida 1977–1986. J Flo Med Assoc.

[5] Bose A, George K, Joseph A (2000). Drowning in childhood: A population based study. Indian Pediatr.

[6] Zhao Z, Svanström L (2003). Injury status and perspectives on developing community safety promotion in China. Health Promot Int.

[7] Ellis AA, Trent RB (1995). Hospitalizations for near drowning in California: Incidence and costs. Am J Public Health.

[8] Jenson LR, Willams SD, Thurman DJ, Keller PA (1992). Submersion injuries in children younger than 5 years in urban utah. West J Med.

[9] Kemp A, Sibert JR (1992). Drowning and near drowning in children in the united kingdom: Lessons for prevention. BMJ.

[10] Pitt WR, Balanda KP (1991). Childhood drowning and near-drowning in Brisbane: The contribution of domestic pools. Med J Aust.

[11] Celis A (1991). Drowning in Jalisco: 1983–1989. Salud Publica Mex.

[12] Kibel SM, Negel FO, Myers J, Cywes S (1990). Childhood near drowning: a 12-year retrospective review. S Afr Med J.

[13] Wintemute GJ (1990). Childhood drowning and near-drowning in the united states. Am J Dis Child.

[14] Hedberg K, Gunderson PD, Vargas C, Osterholm MT, MacDonald KL (1990). Drownings in minnesota, 1980–85: A population-based study. Am J Public Health.

[15] Peden MM, McGee K (2003). The epidemiology of drowning worldwide. Injury Contr Safety Promot.

[16] World Bank (1993). World Development Report 1993: Investing in Health Washington.

[17] World Health Organization (1980). Towards a Better Future: Maternal and Child Health.

[18] Mostafa G, Ahmed K, Shaikh MAK, van Ginneken JK, Sarder AM (1996). Demographic surveillance system—Matlab. V. 27. Registration of demographic events–1995.

[19] Myaux J, Iqbal A, Uzma A, Chakraborty J, Ali M, Hossain M (1996). Environmental hazards as a leading cause of death in children from Bangladesh. Int Child Health.

[20] Baqui AH, Black RE, Arifeen SE, Hill K, Mitra SN, Al Sabir A (1997). Causes of childhood deaths in Bangladesh: results of a nation-wide verbal autopsy study.

[21] Ahmed MK, Rahman M, van Ginneken J (1999). Epidemiology of child deaths due to drowning in matlab. Bangladesh. Int J Epidemiol.

[22] Coffman SP (1991). Parent educations for drowning prevention. J Pediatr Health Care.

[23] Mackie IJ (1999). Patterns of drowning in Australia, 1992–1997. Med J Aust.

[24] Norris B, Wilson JR (2003). Preventing drowning through design—the contribution of human factor. Injury Contr Safety Prom.

[25] Brenner RA (2003). The committee on injury, violence, and poison prevention. Prevention of drowning in infants, children, and adolescents. Pediatrics.

[26] Bull MJ, Agran P, Dowd MD, Garcia V, Gardner HG, Smith GA (2003). (Committee on Injury Violence, and Poison Prevention). Prevention of drowning in infants, children, and adolescents. Pediatrics.

[27] Fergusson DM, Horwood LJ (1984). Risks of drowning in fenced and unfenced domestic swimming pools. N Z Med J.

[28] Pitt WR, Balanda KP (1991). Childhood drowning and near drowning in Brisbane: The contribution of domestic pools. Med J Aust.

[29] Thompson DC, Rivara FP (2000). Pool fencing for preventing drowning in children. Cochrane Database Syst Rev.

[30] Kioeck WG (1993). Infant drowning in nappy buckets. S Afr Med J.

[31] Hyder AA, Arifeen S, Begum N, Fishman S, Baqui AH (2003). Death from drowning: defining a new challenge for child survival in bangladesh. Injury Contr Safety Promot.

[32] Snow RW, Armstrong JRM, Forster D, Winstanley MT, Marsh VM, Newton CRJC (1992). Childhood deaths in Africa: uses and limitations of verbal autopsies. Lancet.

[33] (2002). Health and demographic surveillance system—Matlab. V. 33. Registration of health and demographic events–2000.

[34] Salway SM, Nasim SMA (1994). Levels, trends and causes of mortality in children below 5 years of age in Bangladesh: Findings from a national survey. J Diarrhoeal Dis Res.

[35] Fauveau V, Wojtyniak B, Chakraborty J, Sarder AM, Briend A (1990). The effect of maternal and child health and family planning services on mortality: is prevention enough?. Br Med J.

[36] Brenner RA (2002). Childhood drowning is a global concern: Prevention needs a multifaceted approach (editorial). BMJ.

[37] Brenner RA, Trumble AC, Smith GS, Kessler EP, Overpeck MD (2001). Where children drown, United States, 1995. Pediatrics.

[38] Razzaque A, Nahar L, Sarder AM, van Ginneken JK, Shaikh MAK (1998). Demographic surveillance system—Matlab. V. 29. 1996 Socio-Economic Census.

